# AcoMYB4, an *Ananas comosus* L. MYB Transcription Factor, Functions in Osmotic Stress through Negative Regulation of ABA Signaling

**DOI:** 10.3390/ijms21165727

**Published:** 2020-08-10

**Authors:** Huihuang Chen, Linyi Lai, Lanxin Li, Liping Liu, Bello Hassan Jakada, Youmei Huang, Qing He, Mengnan Chai, Xiaoping Niu, Yuan Qin

**Affiliations:** 1 State Key Laboratory of Ecological Pest Control for Fujian and Taiwan Crops, Key Laboratory of Genetics, Breeding and Multiple Utilization of Crops, Ministry of Education, Fujian Provincial Key Laboratory of Haixia Applied Plant Systems Biology, College of Plant Protection, Fujian Agriculture and Forestry University, Fuzhou 350002, China; huihuangchen@fafu.edu.cn (H.C.); 1170204052@fafu.edu.cn (L.L.); 3170515021@fafu.edu.cn (L.L.); 1180204012@fafu.edu.cn (Y.H.); heqing@fafu.edu.cn (Q.H.); 1170204045@fafu.edu.cn (M.C.); 2 Institute of Science and Technology Austria (IST Austria), Am Campus 1, 3400 Klosterneuburg, Austria; lanxin.li@ist.ac.at; 3 College of Life Science, Fujian Agriculture and Forestry University, Fuzhou 350002, China; bellojakada@gmail.com; 4 State Key Laboratory for Conservation and Utilization of Subtropical Agro-Bioresources, Guangxi Key Lab of Sugarcane Biology, College of Agriculture, Guangxi University, Nanning 530004, China

**Keywords:** pineapple (*Ananas comosus* L.), osmotic stress, ABA, MYB transcription factor

## Abstract

Drought and salt stress are the main environmental cues affecting the survival, development, distribution, and yield of crops worldwide. MYB transcription factors play a crucial role in plants’ biological processes, but the function of pineapple MYB genes is still obscure. In this study, one of the pineapple MYB transcription factors, *AcoMYB4*, was isolated and characterized. The results showed that *AcoMYB4* is localized in the cell nucleus, and its expression is induced by low temperature, drought, salt stress, and hormonal stimulation, especially by abscisic acid (ABA). Overexpression of *AcoMYB4* in rice and Arabidopsis enhanced plant sensitivity to osmotic stress; it led to an increase in the number stomata on leaf surfaces and lower germination rate under salt and drought stress. Furthermore, in *AcoMYB4* OE lines, the membrane oxidation index, free proline, and soluble sugar contents were decreased. In contrast, electrolyte leakage and malondialdehyde (MDA) content increased significantly due to membrane injury, indicating higher sensitivity to drought and salinity stresses. Besides the above, both the expression level and activities of several antioxidant enzymes were decreased, indicating lower antioxidant activity in AcoMYB4 transgenic plants. Moreover, under osmotic stress, overexpression of *AcoMYB4* inhibited ABA biosynthesis through a decrease in the transcription of genes responsible for ABA synthesis (*ABA1* and *ABA2*) and ABA signal transduction factor *ABI5*. These results suggest that AcoMYB4 negatively regulates osmotic stress by attenuating cellular ABA biosynthesis and signal transduction pathways.

## 1. Introduction

As sessile organisms, plants cannot escape adverse environmental conditions like animals can. Biotic and abiotic stresses such as salt stress, drought, heavy metal ion toxicity, and extreme temperature affect not only plant growth and development but also productivity and geographical distribution. To cope with these stresses, plants have evolved elaborate mechanisms ranging from the perception of various stress signals to the modification of physiological and biochemical responses [1]. Meanwhile, plant cells are undergoing numerous changes by regulating a multitude of stress-responsive genes, which can be mainly classified into effectors and regulators based on their functional products [2]. Among them, transcription factors play a dominant role in orchestrating stress signals and functional gene expression, shielding plants from damage resulting from stress [3]. Therefore, genetic engineering of stress-related transcription factors has been proposed to be a promising strategy to improve the stress tolerance of crops for the sustainable development of agricultural production and planting environment [4].

Plants respond to environmental stress through several types of signaling such as abscisic acid (ABA) signaling, reactive oxygen species (ROS) kinases, phospholipids, reactive nitrogen species, calcium, and several other proteins and second messengers that cross-talk with each other. The ABA pathway includes ABA biosynthesis and ABA signal transduction [5,6]. Previous studies showed that a significant number of transcription factors act as ABA-dependent or ABA-independent in response to abiotic stress; they recognize ABA-responsive elements in the promoters of ABA-inducible genes [7,8]. They also activate the expression of target genes responsible for osmo-protection and metabolism [4,9]. ABA production was found to be inhibited in the mutants of crucial enzymes for ABA biosynthesis, ABA deficient 1, and ABA deficient 2 (*ABA1* and *ABA2*) [10,11]. Genetics and protein–protein interaction studies have confirmed that the PYR/PYL/RCAR is the receptor and type A PP2Cs act as co-receptors in the ABA signal transduction [12,13]. PP2Cs negatively regulate ABA response by dephosphorylation and inhibition of SnRK2s kinases. [12]. SnRK2s kinase phosphorylates and activates ABFs/AREBs, including ABA insensitive 3 (ABI3), ABI4, and ABI5 [14]. ABFs binds to ABRE, the promoter of the ABA response, and induces ABA to regulate gene expression, thus controlling seed germination and seedling development [15]. Besides the above, oxidative damage is also a well-known type of stress arising from the metabolism imbalance of ROS. The ROS balance mechanisms have been proven to protect plants from damage caused by drought, salinity, and osmotic stresses [16]. Several studies demonstrated that ROS-scavenging enzymes could improve photosynthesis under hyperosmotic conditions [9,17] and many ROS response genes also involved in antioxidant mechanisms [9,18]. Four enzymes, superoxide dismutase (SOD), catalase (CAT), ascorbate peroxidase (APX), and peroxidase (POD), play roles in the scavenging of ROS [19,20,21]. To elaborate, SOD first catalyzes O_2_^−^ to produce oxygen and H_2_O_2_, and then APX, CAT, and POD metabolize H_2_O_2_ to H_2_O through synergistic action [17,20,22,23]. On the other hand, ROS acts as an important signal molecule involved in plant response to stimuli, having a close relationship with phytohormone ABA [24,25,26,27]. 

The MYB domain-containing proteins comprise a large transcription factor family in plants. MYB proteins have one to four imperfect repeats at the N-terminus [28]. Each repeat consists of 50–53 amino acid residues [29] specifically combined with the motif PyAAC^G^/_T_G [30,31]. Based on the number of imperfect adjacent repeats, MYB proteins are classified into four major subgroups, R1R2R3-type MYB, R2R3-type MYB, MYB1R, and 4R-like MYB proteins. The majority of plant *MYB* genes belong to the R2R3 type and can activate or repress transcription through directly binding to the core sequence in the promoters of the target genes [14]. In previous studies, extensive data revealed that the MYBs form complex webs to modulate a set of physiological and biological processes, including organ development [32,33], stomatal aperture [34], primary and secondary metabolism [32], and hormone stimulated developmental regulation [32,35]. Furthermore, many studies demonstrated that the MYB genes also play a prominent role in abiotic stress response in various species such as *Arabidopsis* [36], rice [37], soybean [38], and poplar [39]. However, the function of MYBs in pineapple is yet to be explored. In this study, a new R2/R3 MYB transcription factor, *AcoMYB4*, was functionally identified as a negative regulator in response to salt and drought stress. Specifically, AcoMYB4 negatively regulates ABA biosynthesis through directly binding to the *AcoABA1* promoter and is negatively involved in ABA signal transduction by regulating expression of *AcoABI5*. 

## 2. Results 

**Figure 1:**
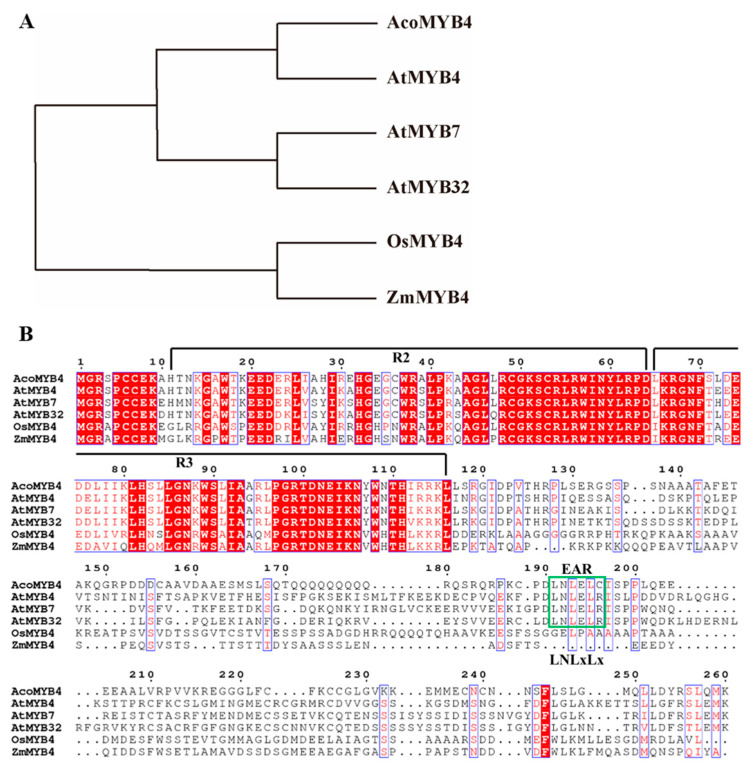
Phylogenetic analysis and multiple alignments of MYBs. Phylogenetic analysis and multiple alignments of MYB family proteins from pineapple, *Arabidopsis*, rice, and zea mays. (**A**) Phylogenetic tree analysis of AcoMYB4, AtMYB4/7/32, OsMYB4, and ZmMYB4. (**B**) Multiple alignments of the conserved R2/R3 domain and EAR motif between AcoMYB4, AtMYB4/7/32, OsMYB4, and ZmMYB4.

Identification and bioinformatics analysis of AcoMYB4A 909 bp cDNA sequence of *AcoMYB4* (Aco013105) was obtained from the pineapple database (http://pineapple.angiosperms.org/pineapple/html/index.html). Sequence analysis showed that it is a full-length sequence with a complete ORF (Open Reading Frame), encoding a peptide of 255 amino acids with a molecular mass of 28.71 kDa and an isoelectric point of 8.17. Homology analysis indicated that this gene shared identity with AtMYB4, ATMYB7, and AtMYB32. Similarly, phylogenetic tree analysis demonstrated that *AcoMYB4* was also a member of the S4 subgroup [40], identified with AtMYB4 (Figure 1A); therefore, it was designated as AcoMYB4. Multiple alignments showed that AcoMYB4 shared a highly conserved R2/R3 domain with highly conserved tryptophan residues. Besides the above, AcoMYB4 shared an EAR domain, LNL^D^/_E_Lxi^G^/_S_ repression motif, at the C-terminal with three Arabidopsis MYBs, AtMYB4, AtMYB7, and a repression motif which functions as a negative regulate in response to abiotic stress (Figure 1B). This result indicates that AcoMYB4 possesses a negative function in response to abiotic stress. 

### 2.1. Expression Profiles of AcoMYB4 Response to Various Abiotic Stresses

**Figure 2:**
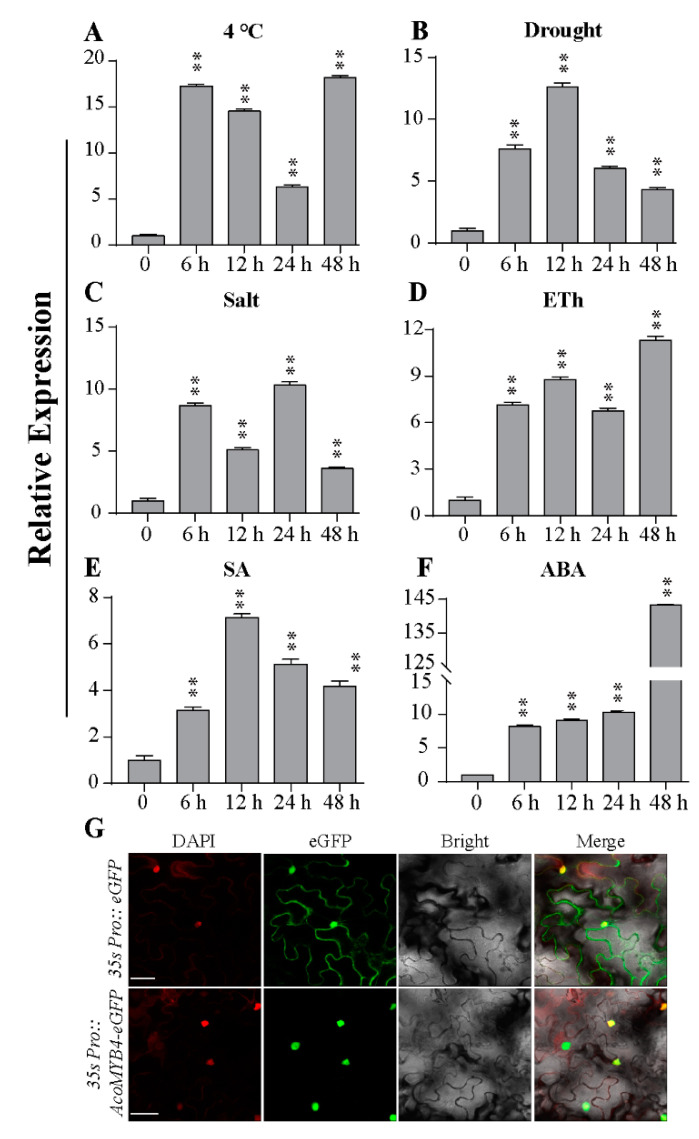
Expression patterns and subcellular localization of AcoMYB4. Relative expression levels of *AcoMYB4* under different treatments: (**A**) cold, (**B**) drought, (**C**) salt, (**D**) Eth, (**E**) SA, and (**F**) ABA. For each treatment, the expression level at 0 h was set as 1.0 and values are means of three replicates. (**G**) AcoMYB4 subcellular localization was shown in *Nicotiana Benthamiana* leaves’ surface cells, Bar = 5 μm. The error bars indicate + SD (*n* = 3). Asterisks indicate significant differences for the indicated comparisons based on Student’s *t*-test (** *p* < 0.01).

*AcoMYB4* expression patterns indicated that it may involve an abiotic stress response. The transcript level of AcoMYB4 was detected under various stresses by qRT-PCR to evaluate the function of AcoMYB4 in stress response. Our results showed that AcoMYB4 responded positively to cold, drought, salt, ethephon (Eth), salicylic acid (SA), and ABA (Figure 2A–F). Specifically, the *AcoMYB4* transcripts increased dramatically to 17-fold at 6 h after cold stimulation, followed by a gradual decline and return to a maximum level at 48 h (Figure 2A). Under drought and SA treatments, *AcoMYB4* mRNA accumulated and reached a maximum level of 12 h (Figure 2B,E). Meanwhle, under salt stress, the transcript level of *AcoMYB4* increased significantly at 6 h, followed by a decrease, and reached its maximum at 24 h, afterwards declining again at 48 h (Figure 2C). Eth and ABA stimulation induced *AcoMYB4* transcripts and reached a maximum level after 48 h (Figure 2D,F). AcoMYB4 expression patterns indicated that it may involve an abiotic stress response.

### 2.2. Subcellular Localization of AcoMYB4

For subcellular localization of AcoMYB4, full-length CDS (Coding Domain Sequence) of AcoMYB4 was fused to N-terminal of GFP (Green Fluorescent Proteins) reporter protein driven by CaMV 35S promoter, generating a fusion protein AcoMYB4-GFP, and the empty vector with GFP alone was used as control. The GFP signals were analyzed in *N. benthamiana* epidermal cells via Agrobacterium-mediated transient transformation. Microscopic visualization showed that the AcoMYB4-GFP protein was exclusively detected in the nucleus (Figure 2G) and could overlap with DAPI (4′, 6-diamidino-2-phenylindole) signals, whereas the control GFP signals were uniformly distributed throughout the whole cell (Figure 2G). These results suggest that AcoMYB4 is a nucleus-localized protein.

### 2.3. Overexpression of AcoMYB4 Plants Was Sensitive to Drought and Salt Stress

As the transcripts of *AcoMYB4* were strongly induced by drought and salt stress, we speculated that AcoMYB4 may be involved in drought and salinity stresses. To test this hypothesis, we generated transgenic rice and *Arabidopsis* plants overexpressing *AcoMYB4*. All the positive transgenic lines were screened by Basta and further validated by PCR analysis. Two transgenic rice (designated as OsOE-28# and OsOE-31#) and *Arabidopsis* (designated as AtOE-3# and AtOE-16#) lines with high transcript levels of *AcoMYB4* and not disturbing the expression of endogenous genes were selected for further experiments (Figure S1A,B). 

**Figure 3:**
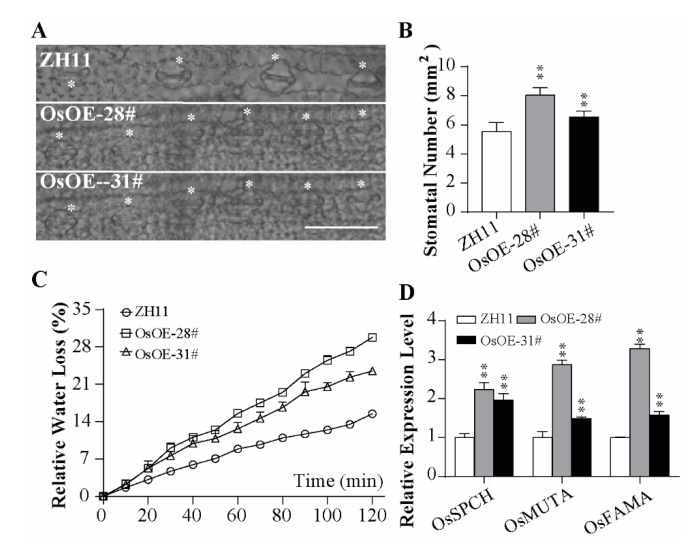
Characteristics of the stomatal phenotype of *AcoMYB4* overexpression lines. (**A**,**B**) Stomatal number on the epidermis of underside of leaves at 5 leaf age of ZH11, OsOE-28#, and OsOE-31#; the error bars indicate + SD (*n* = 20), Bar = 1 mm. (**C**) Relative water loss of detective leaves during a 120-min dehydration; the error bars indicate + SD (*n* = 3). (**D**) Relative expression level of rice stomatal development related genes; the error bars indicate + SD (*n* = 3). White “*” indicates the location of the stomata. Asterisks indicate significant differences for the indicated comparisons based on Student’s *t*-test (** *p* < 0.01; * *p* < 0.05).

Stomata is an essential indicator of water loss. Specifically, higher stomata numbers per square millimeter were found in overexpression lines compared to the wild type (1.15-fold in OsOE-28# and 1.18-fold in OsOE-31#; 1.62-fold in AtOE-3# and 1.45-fold in AtOE-16#) (Figure 3A,B; Figure S2B,C, and Table S1). In line with the stated result, the water loss amounts in 2 h in OsOE-28# and OsOE-31# were around 1.83 times and 1.57 times more compared to the wild type (Figure 3C, Figure S2A, and Table S1). Furthermore, we investigated the transcription levels of the genes, Os*FAMA*, Os*MUTE*, and Os*SPCH*, controlling stomatal development [41,42]. The results showed that the expression levels of these genes were significantly higher in the rice OE lines (ranging from 1.50 to 3.30-fold) than the wild type, while one gene was increased and the others decreased in the *Arabidopsis* OE lines (Figure 3D, Figure S2D, and Table S1). 

**Figure 4:**
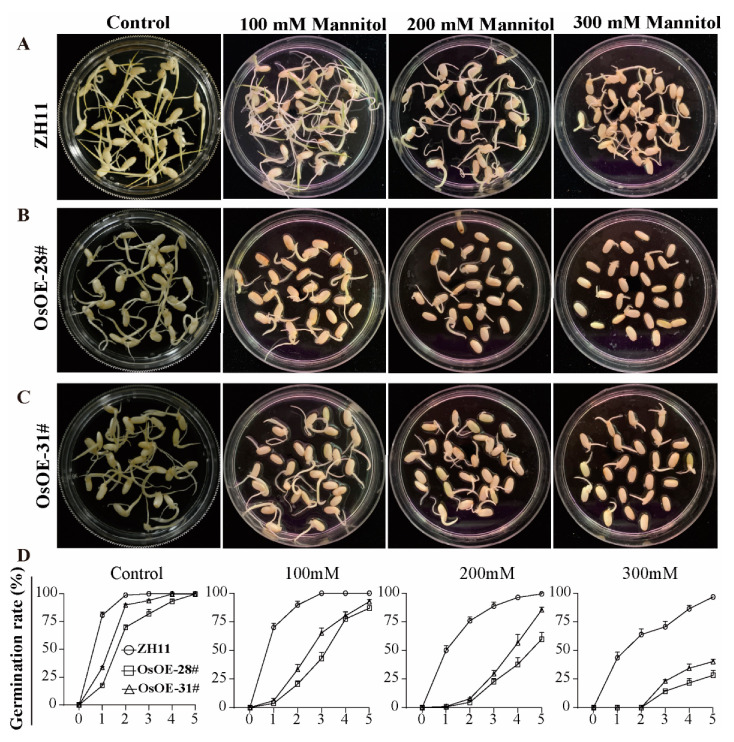
*AcoMYB4* overexpression lines seed germination rate under mannitol treatment. Transgenic rice seeds were grown on media containing different mannitol concentrations. Photographs show seedlings of transgenic rice (**A**–**C**; 5th day) grown on different mediums at the end of stratification. (**D**) Transgenic rice seed germination rates were quantified from the first to last day after sowing. The error bars indicate + SD (*n* = 3 replicates).

To further investigate the role of AcoMYB4 in abiotic stress, we analyzed the germination rate of the OE lines. Under normal conditions, though AtOE-3# and AtOE-16# germinated one day later than WT (Wild Type) at the beginning (Figure S3B), the maximum germination rate (after the third day) and cotyledon greening rate (after fifth day) of AtOE lines were similar to WT (Figure S3). Nonetheless, with the increase in mannitol concentration, the germination rate of AtOE lines (around 12.00%) were significantly decreased compared with WT (Figure S3B; Table S1). Moreover, under a high concentration of mannitol (400 mM), WT had a cotyledon greening rate of nearly 13.70% at the ninth day; in comparison, almost none of the germinated AtOE seeds could enter the cotyledon stage (Figure S3C). The germination process of OsOE lines was four days later than that of WT, whereas all the seeds were able to germinate completely after 5 days (Figure 4). Similarly, both AtOE and OsOE lines had a dramatic decrease shown in the germination rate after salinity stimulation, and OsOE lines had an additionally reduced germinate rate after dehydration (Figure 4, Figures S4 and S5). In summary, overexpression of AcoMYB4 decreased the tolerance of plants in response to drought and salinity stress.

### 2.4. Overexpression of AcoMYB4 Decreased Antioxidant Enzyme Activities and Enhanced Oxidative Damage

**Figure 5:**
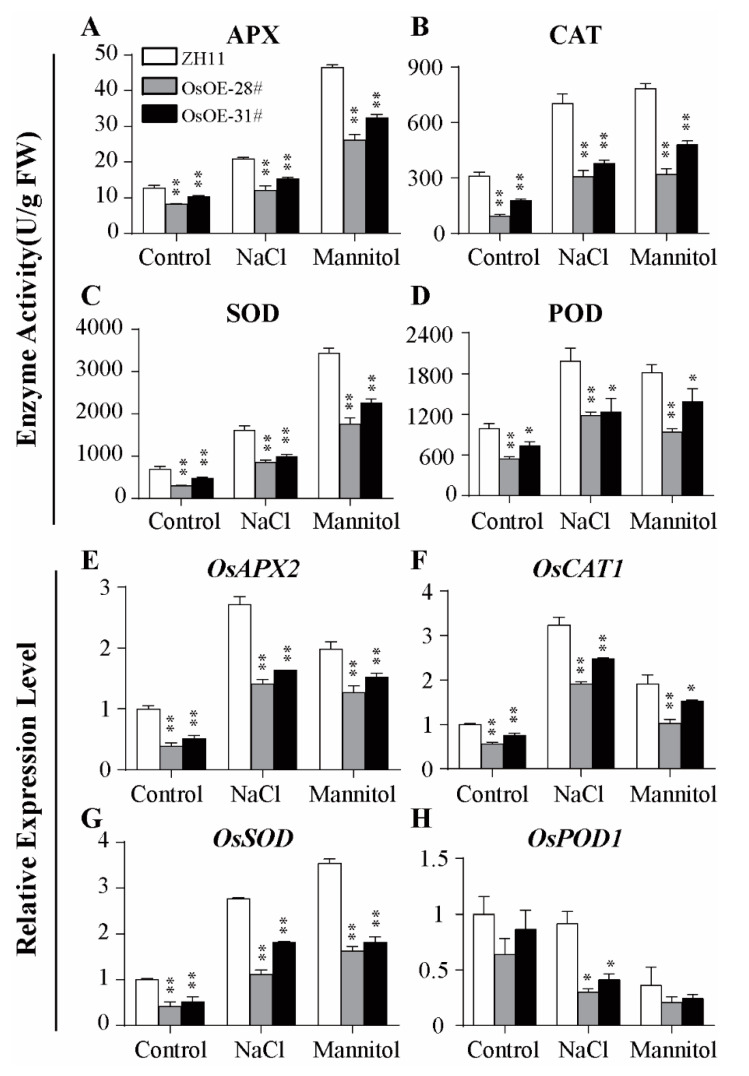
ROS scavenges enzyme activities of *AcoMYB4* transgenic rice lines under osmotic stress. The water-cultured seedlings of OsOE-28#, OsOE-31#, and ZH11 at five-leaf age were transferred to a nutrient solution with or without 150 mM NaCl and 200 mM mannitol for 2 days. (**A**) APX, (**B**) CAT, (**C**) SOD, and (**D**) POD enzyme activity were directly determined from fresh leaves. The relative expression levels of (**E**) *OsAPX2*, (**F**) *OsCAT1*, (**G**) *OsSOD*, and (**H**) *OsPOD1* were analyzed by qRT-PCR. The error bars indicate + SD (*n* = 3 replicates). Asterisks indicate significant differences for the indicated comparisons based on Student’s *t*-test (** *p* < 0.01; * *p* < 0.05).

To evaluate the stress tolerance of OE lines under salt or drought treatment, we analyzed the activity of ROS scavenging enzymes such as APX, CAT, SOD, and POD [22] and the expression levels of corresponding genes (*APX2*, *CAT1*, *SOD*, and *POD1*). The enzyme activities of OsOE lines were significantly lower than those of WT lines when the plants were exposed to osmotic stress (Figure 5A–D). Consistent with the enzyme activities, the mRNA levels of *APX*, *CAT*, *SOD*, and *POD* were also dramatically decreased in both AtOE and OsOE lines (Figure 5E–H and Figure S6).

**Figure 6:**
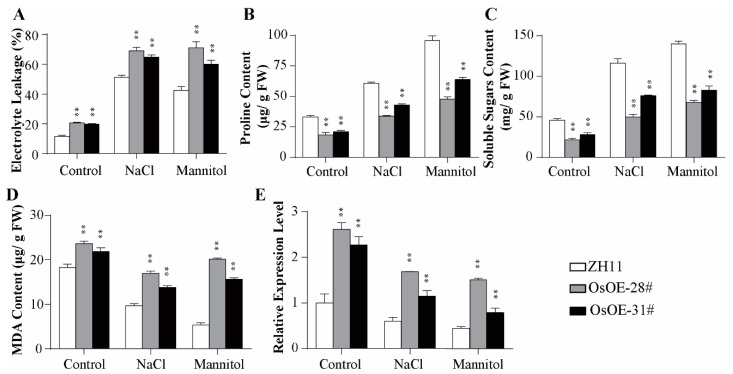
Oxidative damage of *AcoMYB4* transgenic rice lines under osmotic stress. (**A**) Electrolyte leakage, (**B**) proline, (**C**) soluble sugars, and (**D**) MDA content as well as (**E**) *OsMDA4* relative expression level in wild types and transgenic lines under salt or drought treatment for 2 days. The error bars indicate + SD (*n* = 3 replicates). Asterisks indicate significant differences for the indicated comparisons based on Student’s *t*-test (** *p* < 0.01).

In addition, we analyzed electrolyte leakage, an important indicator of membrane injury, in the OE lines. The rice OE lines had higher electrolyte leakage than WT lines under salt and drought treatments, suggesting that the *AcoMYB4* transgenic lines suffered from severe membrane damage. These results prompted us to investigate the membrane oxidation damage indexes (the content of free proline, soluble sugars, and MDA) in WT and the transgenic lines under drought or salt condition. We found that the OE lines have a lower amount of free proline (Figure 6B; Figure S7B) and soluble sugars (Figure 6C; Figure S7C), while having higher MDA content and expression levels of corresponding gene *MDA4* (Figure 6D,E; Figure S7D,E). These results further demonstrate that the transgenic lines were more sensitive to drought and salinity stress through membrane injury. 

### 2.5. AcoMYB4 Regulates ABA Biosynthesis by Directly Binding to the Promoters of AcoABI5 and AcoABA1

**Figure 7:**
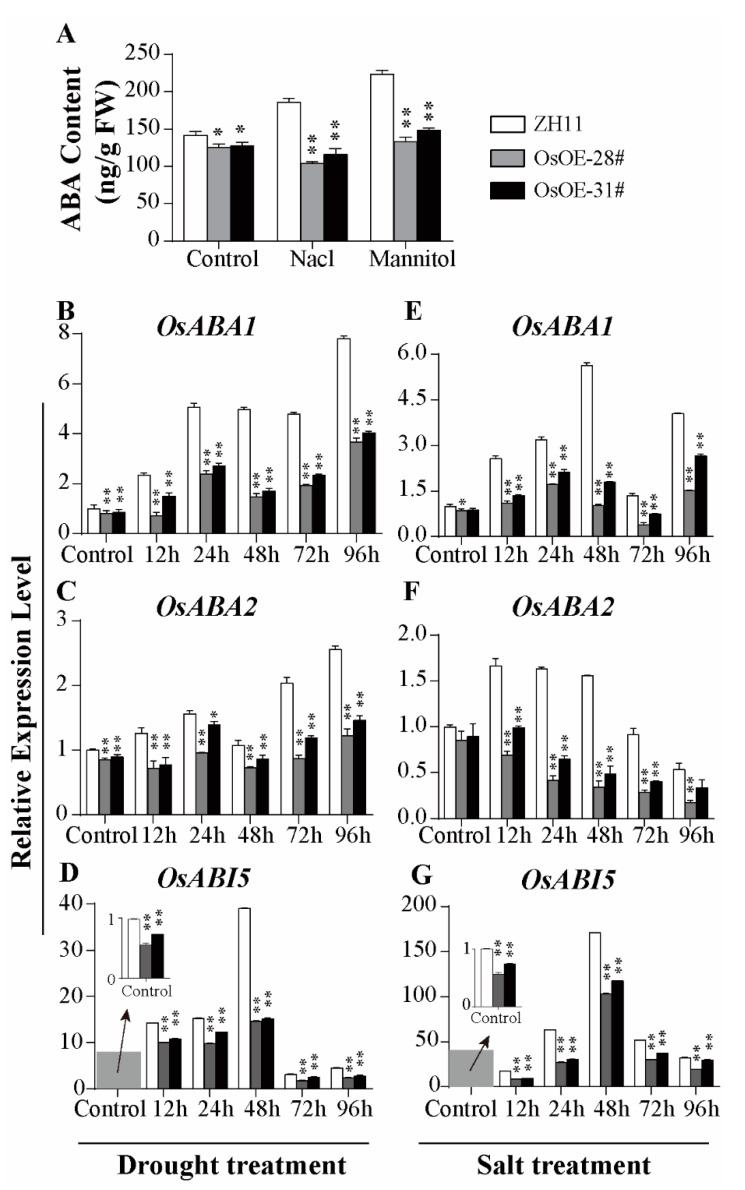
Determination of ABA content and expression level of ABA signal pathway genes in transgenic rice lines. (**A**) The endogenous ABA levels of transgenic rice lines and wild-type leaves were determined by LC-MS under normal, salt, or drought conditions for 48 h, respectively. The expression levels of *OsABA1*, *OsABA2*, and *OsABI5* in WT, OsOE-28#, and OsOE-31# under (**B**–**D**) mannitol or (**E**–**G**) NaCl treatment. The error bars indicate + SD (*n* = 3 replicates). Asterisks indicate significant differences for the indicated comparisons based on Student’s *t*-test (** *p* < 0.01; * *p* < 0.05).

ABA, an important endogenous phytohormone, plays a crucial role in the response to osmotic stress [11]. As reported, osmotic stress induces ABA biosynthesis, and the increase in ABA level has a positive feedback effect on the ABA biosynthesis pathway [11]. Hence, further work was carried out to analyze whether the ABA synthesis was altered in the *AcoMYB4* transgenic plants. LC-MS was employed to analyze the ABA content in these tested plants and WT lines. As shown in Figure 7A and Figure S8A, the ABA content of OE lines (around 88%–92%) were lower than that of WT under normal conditions, and the decrease was more obvious, amounting to 56%–66% in OsOE lines and 44%–85% in AtOE lines, under salt or drought stresses (Table S1). This coincides with the lower transcriptional levels of *ABA1* and *ABA2* in the transgenic rice lines (Figure 7B,C,E,F). Similar results were also observed in the transgenic *Arabidopsis* lines, although with a slight difference in the transcripts of *AtABA2* (Figure S8B,C,E,F). The expression level of *ABI5*, an ABA-insensitive factor of the ABA signal pathway [43,44], was decreased in normal conditions in OE lines, while it was dramatically induced in stress treatment in ZH11, two-fold more than OsOE lines (Figure 7D,G; Figure S8D,G). 

**Figure 8:**
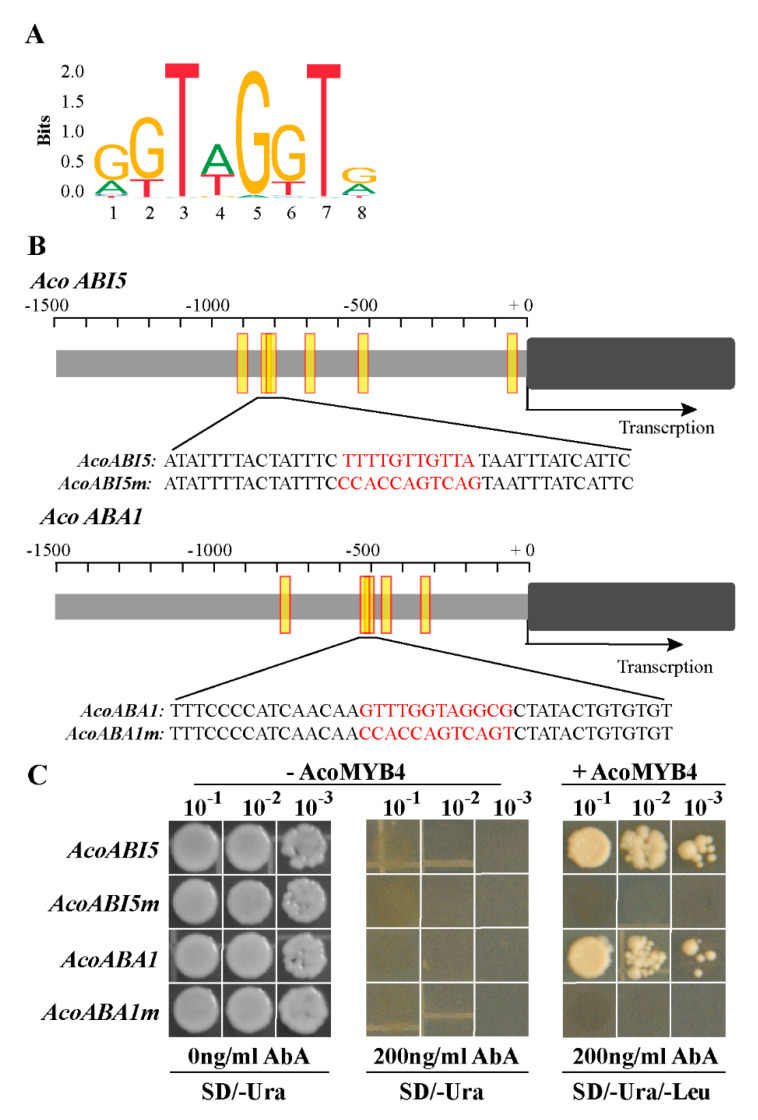
AcoMYB4 binds to the promoter of *AcoABI5* and *AcoABA1*. (**A**) The binding sequence logo of MYB4 downloaded from JASPAR. (**B**) The binding site of MYB4 was predicted to be in the cis-elements of *AcoABI5* and *AcoABA1* and the magnified region shows the specific binding site and its mutation sequence (*AcoABI5m* and *AcoABA1m*). The yellow box indicates the location of the binding site. (**C**) Yeast one-hybrid assay using vectors contains MYB4 binding site or fragments with introduced mutations. Yeast cells carting or lacking pGAD-AcoMYB4 were grown on SD/-Ura or SD/-Ura, SD/-Ura/-Leu containing 200 ng/mL AbA.

These results implied that the ABA synthesis genes might be the potential targets that are regulated by AcoMYB4. To verify this hypothesis, around 1000 bp promoters of *AcoABI5* and *AcoABA1* were isolated by genomic PCR. Bioinformatics analysis (http://jaspar.genereg.net/) indicated that the promoters of *AcoABI5* and *AcoABA1* contained putative MYB binding motifs (Figure 8A,B). In detail, the promoter sequence of *AcoABI5* and *AcoABA1* contains six and five potential binding elements, respectively. Moreover, there were two overlapping elements between *AcoABI5* and *AcoABA1* and only 4 bp base differences (Table S2). Therefore, a yeast one-hybrid assay was adopted to investigate whether AcoMYB4 could bind to these elements of *AcoABI5* and *AcoABA1* promoter. A 70 bp normal fragments and fragments with introduced mutations (named *AcoABI5m* and *AcoABA1m*) containing the MYB binding elements were used as bait and cloned into the pABAi vector (Figure 8B), while AcoMYB4 was used as prey. As shown in Figure 8C, the yeast cells of normal samples and fragments with introduced mutations groups grew well on the screening medium (SD/-Ura). However, cell growth of fragments with introduced mutations groups was completely inhibited by 200 ng/mL AbA, while cells of AcoMYB4 that were transformed into the yeast survived (Figure 8C). These results suggest that AcoMYB4 can directly bind to the promoters of *AcoABI5* and *AcoABA1* in yeast. 

## 3. Discussion

MYB transcription factors, one of the largest plant transcription factor families, play central roles in plants’ responses to various biotic [45] and abiotic stresses [46]. Although some MYBs have been characterized [47], the biological functions of most plant MYBs are still unclear, particularly in those non-model plants. Therefore, characterization of the function and mechanism of MYB TFs in pineapple will contribute to the current knowledge of the roles of MYBs in pineapple development and stress response. In this study, we characterized the R2R3-type MYB transcription factor *AcoMYB4* from pineapple. AcoMYB4 possessed the signature motifs defining a typical R2R3-type MYB, and multiple alignments revealed that AcoMYB4 shared high identity with AtMYB4, AtMYB7, and AtMYB32, indicating that *AcoMYB4* is a putative homologue of pineapple.

The transcripts of *AcoMYB4* were induced by drought and salinity, indicating that *AcoMYB4* might be involved in osmotic stress. Therefore, AcoMYB4 was ectopically expressed in rice and *Arabidopsis* to elucidate its function under drought and salinity conditions. The OE plants displayed greater susceptibility to drought and salinity, as indicated by the higher rate of water loss, higher levels of MDA, low content of free proline and soluble sugar, and more stomatal numbers than WT plants. As MDA levels and electrolyte leakage determine membrane peroxidation, it is evident that the transgenic plants showed a higher degree of membrane injury under stress conditions than WT plants. Previous studies revealed that lipid peroxidation mostly resulted from excessive accumulation of ROS. To detoxify stress-induced ROS, plants have evolved a delicate system for ROS detoxification by ROS-scavenging enzymes (APX, CAT, POD, and SOD) [17,27]. Our results showed lower enzyme activities of OE lines than WT. The corresponding genes of the enzymes also exhibited lower transcript levels than WT (Figure 5, Figure S6). This result is consistent with the ROS-associated membrane damage caused by electrolyte leakage and MDA content (Figure 6D; Figure S7D), indicating that AcoMYB4 played a negative role in drought and salinity stress. The MYB factors belonging to the same subgroup are functionally conservative in some places. This is the case in subgroup 4, which includes *AtMYB4*, A*tMYB7*, and *AtMYB32* as transcriptional suppressors [48]. It has been observed that *AtMYB4*, *AtMYB7*, and *AtMYB32* play a negative regulatory role in the phenylpropanoid pathway. However, their functions are also divided. *AtMYB32* alone participates in pollen development, *AtMYB7* responds to salt stress [49], and *AtMYB4* does not participate in these biological processes. *AtMYB4* not only plays a negative role in flavonid biosynthesis but also plays a positive role in response to cadmium stress [50]. This may be associated with the differences in their temporary and spatial expressions. Therefore, the functional characterization of *AcoMYB4*, which also belongs to the S4 subfamily, also needs more extensive research in order to elaborate its extensive signal regulatory networks.

ABA, an endogenous hormone, is essential to the plant adaptive response, and it is strongly induced by osmotic signals to protect plants from adverse effects of the environment [51]. ABA is involved in ROS production and scavenging via transcriptional regulation; it induces the expression of antioxidant genes including SOD, CAT, and APX. It enhances the activity of these antioxidant enzymes to reduce the oxidative damage of ROS [20]. In *Arabidopsis*, ABA synthesis-deficient mutants showed reduced tolerance to osmotic stress [52]. To understand the regulatory function of AcoMYB4, we checked the transcript levels of genes involved in ABA biosynthesis (*ABA1* and *ABA2*) and antioxidant genes (*APX*, *CAT*, *POD*, and *SOD*). The RT-qPCR results showed that the expression levels of these genes were lower in the transgenic plants compared to WT plants under stress conditions. Interestingly, the transcript levels of two genes (*ABA1* and *ABA2*) involved in ABA biosynthesis were significantly reduced compared to the WT plants under osmotic stress (Figure 7B,C,E,F; Figure S8B,C,E,F). Furthermore, ABI5 plays a positive regulatory role in osmotic stress [44], and the transcript levels of this gene in the transgenic plants were lower than those of the WT lines (Figure 7D,G; Figure S8D,G). Moreover, AcoMYB4 could bind the promoter of *AcoABA1* and *AcoABI5*, indicating that *AcoABA1* and *AcoABI5* were the target genes of AcoMYB4 (Figure 8C). All these results demonstrate that AcoMYB4 plays a negative role in osmotic stress through reducing the content of ABA. 

Increased crop yield can be achieved via agricultural irrigation, water-use efficiency (WUE), and harvest index. WUE may be regulated not only by a small stomatal aperture but also by increasing photosynthetic capacity in C3 plants [53]. It has been well established that increased ABA content triggers stomatal closure [54], thus enhancing WUE. ABA content in OE lines was significantly reduced compared to the wild type under normal or stress conditions (Figure 7A; Figure S8A). Due to our technical limitations, we only observed more stomatal numbers (Figure 3A,B) in rice leaves and did not detect a stomatal aperture. In addition, whether the faster relative water loss rates in OE lines (Figure 3C) were associated with ABA-triggered stomatal closure should be further identified.

In summary, we characterized a novel osmotic stress response MYB transcription factor AcoMYB4 in pineapple, which played a negative role in salt and drought tolerance. The transcript level of *AcoMYB4* was regulated by osmotic stress and ABA stimulation. Overexpression of *AcoMYB4* lines have more stomata on their leaf surface and reduce the activity of antioxidant enzymes. Bioinformatics analysis showed that ABA synthesis genes possess MYB binding elements, indicating that genes involved in ABA biosynthesis may be the direct target of AcoMYB4. The Y1H assay supported a direct interaction between AcoMYB4 and the promoter of *AcoABA1* and *AcoABI5*. All in all, these results indicate that AcoMYB4 plays a crucial role in osmotic stress via the ABA pathway. However, much more work needs to be conducted in order to decipher other components that interact with AcoMYB4 in the future to obtain a better understanding of the molecular mechanisms underlying AcoMYB4 under osmotic stress.

## 4. Materials and Methods

### 4.1. Plant Materials and Stress Treatment of Pineapple

Pineapple (*A. comosus* var MD-2) was provided by Haixia Institute of Science and Technology Centre for Genomics and Biotechnology, Fujian Agriculture and Forestry University, Fuzhou, China. *Arabidopsis thaliana* Columbia ecotype and *Oryza sativa* L. plants ZH11 (Zhong Hua 11) were used as wild-type plants. *AcoMYB4* transgenic *Arabidopsis* and rice lines were obtained through *Agrobacterium tumefaciens*-mediated transformation [55,56]. Two lines of T2 generation seeds with a high *AcoMYB4* transcription level were used for further analysis. 

Ninety-day-old pineapple seedlings were selected for osmotic stress treatment and hormone stimulation. Specifically, the roots of the seedlings were soaked in 200 mM sodium chloride, 20% PEG6000 (Polyethyleneglycol, M/V), 5.0 mM ethephon (Eth), 100 μM abscisic acid (ABA), and 100 μM salicylic acid (SA) solutions, respectively. For the low temperature treatment, the seedlings were placed in the artificial climate chamber at 4 °C and a normal growth temperature, as described by Bartholomew [57]. The experiments were repeated at least three times with three independent biological replicates. 

### 4.2. Bioinformatics Analysis

The full length of protein and CDS were downloaded from the pineapple database (http://pineapple.angiosperms.org/pineapple/html/index.html). The primer sequence used in this work is listed in Supplementary Table S3. The phylogenetic tree was generated using the maximum likelihood module of PhyloSuite software [58]. Homologous protein sequences were downloaded from Phytozome, and accession numbers of those sequences are as follows: AtMYB4 (AT4G38620), AtMYB7 (AT2G16720), AtMYB32 (AT4G34990), OsMYB4 (LOC_Os10g33810), and ZmMYB4 (GRM_ZM2G095904). Multiple sequence alignment was performed by ESPript3.0 [59].

### 4.3. RNA Extraction and qRT-PCR

Samples were collected at 0, 6, 12, 24, and 48 h post-treatment, and three independent seedlings were randomly harvested and frozen by liquid nitrogen immediately, then stored at −80 °C store for RNA extraction. Total RNA was extracted using the OMEGA Plant RNA Kit (R6827), following the manufacturer’s instructions. Takara’s PrimeScript Synthesis 1st Strand cDNA Synthesis Kit (6110B) was used for the cDNA synthesis. Quantitative real-time PCR analysis was performed using TransStart^®^ Top Green qPCR SuperMix (TransGen, AQ132-11) at a 20 μL volume and Bio-Rad CFX-96 detection system with the following steps: 94 °C for 30 s; then, 40 cycles of 94 °C for 5 s, 60 °C for 15 s, and 72 °C for 10 s; followed by a melt cycle from 65 °C to 95 °C. Pineapple *AcoPP2A* [60], *Arabidopsis AtACT2*, and rice *OsUBQ5* were selected as reference genes, respectively. All the experiments were carried out in three technical and three biological replicates.

### 4.4. Cloning and Subcellular Localization

The fragment of *AcoMYB4* was amplified from pineapple cDNA using primer MYB4CDS-F and MYB4CDS-R. *AcoMYB4* was then constructed into pENTR™/D-TOPO vector (CAT: K2400-20, Invitrogen). Afterwards, positive clones were recombined with dual carrier pGWB605 (Invitrogen) by LR clone II enzyme (Invitrogen) and then transformed into *Agrobacterium tumefaciens* GV3101. The construct was then used for subcellular localization. Fused *AcoMYB4::GFP* was driven by CaMV 35S promoter fused with a green fluorescent protein (GFP) and *35S::GFP* empty vector was used as a negative control. The construct was suspended in an infection buffer (10 mM MES, 50 mM MgCl_2_, pH = 5.8, 100 μm AcetoSyringone), and it was then injected into the leaf of *Nicotiana benthamiana*. After dark growth for 36 or 48 h, DAPI (4′,6-diamidino-2-phenylindole) was injected into the leaf as a nucleus marker. GFP signal was observed using LAICA SP8 confocal microscope, with a 488 nm wavelength for GFP signal and a 405 nm wavelength for the DAPI signal.

### 4.5. Germination, Cotyledon Greening, and Survival Rate of Transgenic Plants

Seeds of wild-type and overexpressed lines were collected at the same time and were used to analyze the germination and cotyledon greening rate. In the follow-up, except for special instructions, 3-week-old *Arabidopsis* and 5-leaf-old rice materials were used for analysis and treatment.

*Arabidopsis* seeds were germinated on a plate containing ½ Murashige and Skoog medium. Volumes of 200, 300, and 400 mM mannitol were used to simulate drought treatment, and 100, 150, and 200 mM NaCl were used to mimic salt treatment, while the control group did not receive any stress treatment. Rice seeds without glumes were germinated in the nutrient solution [61]. In detail, the nutrient solutions containing 100, 200, and 300 mm mannitol or 100, 150, and 200 mM NaCl were used to simulate drought and salt stress, respectively. Each treatment was repeated three times, and in each line, more than 100 seeds were used and their germination rate was counted every day. The detailed statistics are shown in Supplementary Table S1. 

### 4.6. Assessment of Drought and Salt Tolerance in Transgenic Plants

The leaves of transgenic seedlings were cut down and placed on dishes containing filter paper, and the fresh weight of leaves was measured per 20 min. The relative water loss rate was calculated compared to the initial weight. The stomatal number was observed according to Li’s description [62]. For the determination of electrolyte leakage, plant material was washed with double distilled water. A total 0.5 g evenly cut leaves (avoiding the main vein) were placed in a tube with 20 mL of double distilled water. After 1 h shaking at room temperature, the electrical conductivity of the solution (L1) was measured. Then, the sample was boiled for 20 min and the electrical conductivity (L2) was measured for the second time. The formula for electrolyte leakage rate is EL (%) = (L1/L2) × 100%.

### 4.7. Analysis of Proline, Soluble Sugars, ABA Content, and Enzyme Activity

The content of free proline (BC0290), soluble sugars (BC0035), and MDA(BC0025), the enzyme activity of POD (BC0090), CAT(BC0205), SOD (BC0175), and APX(BC0220) detection were performed according to the operation manual of Solarbio (Beijing Solarbio Science & Technology, Beijing, China). ABA content was measured by the liquid chromatography tandem mass spectrometry (LC-MS) system (Agilent HPLC-1200 tandem mass spectrometry 6495C). The parameter information refers to Perin’s report [63].

### 4.8. Yeast One-Hybrid System

Through homologous comparison, *AcoABI5* (Aco027121, homologous gene of *AtABI5* and *OsABI5*) and *AcoABA1* (Aco005893, homologous gene of *AtABA1*, *AtABA2*, *OsABA1*, and *OsABA2*) were identified. A 1000 bp sequence upstream of the transcription star site of *AcoABA1* and *AcoABI5* was defined as the promoter region of these two genes. MYB4 binding cis-acting elements on *AcoABA1* and *AcoABI5* promoters were predicted by JASPAR (http://jaspar.genereg.net/). 

*AcoMYB4* CDS without stop codon was amplified and then integrated into the pGADT7-T vector by an in-fusion cloning kit (Clontch, Takara) to form a pGADT7-AcoMYB4 bait report vector. Meanwhile, the predicted normal or mutational fragments were synthesized by DNA synthesis technology (Sangon Biotech, Shanghai, China; Table S2) and cloned into a pABAi vector by in-fusion technology to form pABAi-AcoABA1, pABAi-AcoABA1m, pABAi-AcoABI5, and pABAi-AcoABI5m prey report vectors, respectively. 

Yeast one-hybrid was carried out according to instructions provided by Clontech (Takara). Prey was transformed into Y1H gold yeast strain and cultured on SD/-Ura or SD/-Ura/-Leu medium with or without 200 ng/mL Aureobasidin A (AbA) for 3 days. In addition, the yeast cells co-transformed by prey and bait were cultured on SD/-Ura/-Leu medium containing 200 ng/mL AbA for 3 days.

### 4.9. Statistical Analysis

Experiments were carried out with three biological repeats and three technical repeats, and Y1H was carried out with three technical repeats. Student’s *t*-test was used to analyze the significant differences between each treatment and the appropriate control group by GraphPad Prism 8.0.1 software. Detailed summaries of statistical analysis are shown in Supplementary Table S1. We took *p* < 0.05, *p* < 0.01 to indicate significant differences.

## 5. Conclusions

In this study, we cloned and characterized pineapple *AcoMYB4*. Overexpression of *AcoMYB4* in rice and *Arabidopsis* resulted in more stomatal numbers and decreased tolerance to drought and salinity stress. Our findings revealed that the AcoMYB4-AcoABA1 and AcoMYB4-AcoABI5 signal pathway in pineapple expands our understanding of complex osmotic stress signal networks, which might be crucial for developing environmentally stress-resistant pineapple.

## Supplementary Materials

The following are available online at https://www.mdpi.com/1422-0067/21/16/5727/s1. Figure S1: Analysis of expression level of AcoMYB4 and its homologous genes, Figure S2: Characteristics of stomatal phenotype of AcoMYB4 overexpress *Arabidopsis* lines, Figure S3: Response of AcoMYB4 overexpression plants seed germination and cotyledon greening rate in mannitol stress, Figure S4: AcoMYB4 overexpression lines seed germination rate under NaCl stress, Figure S5: AcoMYB4 overexpression lines seed germination and cotyledon greening rate under NaCl stress, Figure S6: ROS scavenges enzyme activities of AcoMYB4 transgenic *Arabidopsis* lines under salt or drought condition, Figure S7: Oxidative damage of AcoMYB4 transgenic *Arabidopsis* lines under osmotic stress, Figure S8: Determination of ABA content and expression level of ABA signal pathway genes in transgenic *Arabidopsis* lines, Table S1: Detailed statistical and Student’ *t* test analyses in this study, Table S2: Synthetic information of AcoABA1, AcoABI5, AcoABA1m and AcoABI5m fragements, Table S3: The primers used for vector construction and qRT-PCR.
