# Supplementary material for: AcoMYB4, an Ananas comosus L. MYB Transcription Factor, Functions in Osmotic Stress through Negative Regulation of ABA Signaling
Source: Int J Mol Sci. 2020 Aug 10;21(16):5727. doi: 10.3390/ijms21165727 (PMC7460842; doi:10.3390/ijms21165727)
Supplement: Supplementary file 1 [file ijms-21-05727-s001.zip › Supplemental filed/Supplemental Figures .pdf]

AcoMYB4, an *Ananas comosus L.* MYB transcription factor, functions in osmotic stress through negative regulation of ABA signaling

Huihuang Chen, Linyi Lai, Lanxin Li, Liping Liu, Bello Hassan Jakada, Youmei Huang, Qing He, Mengnan Chai, Xiaoping Niu and Yuan Qin

Supplemental Figures

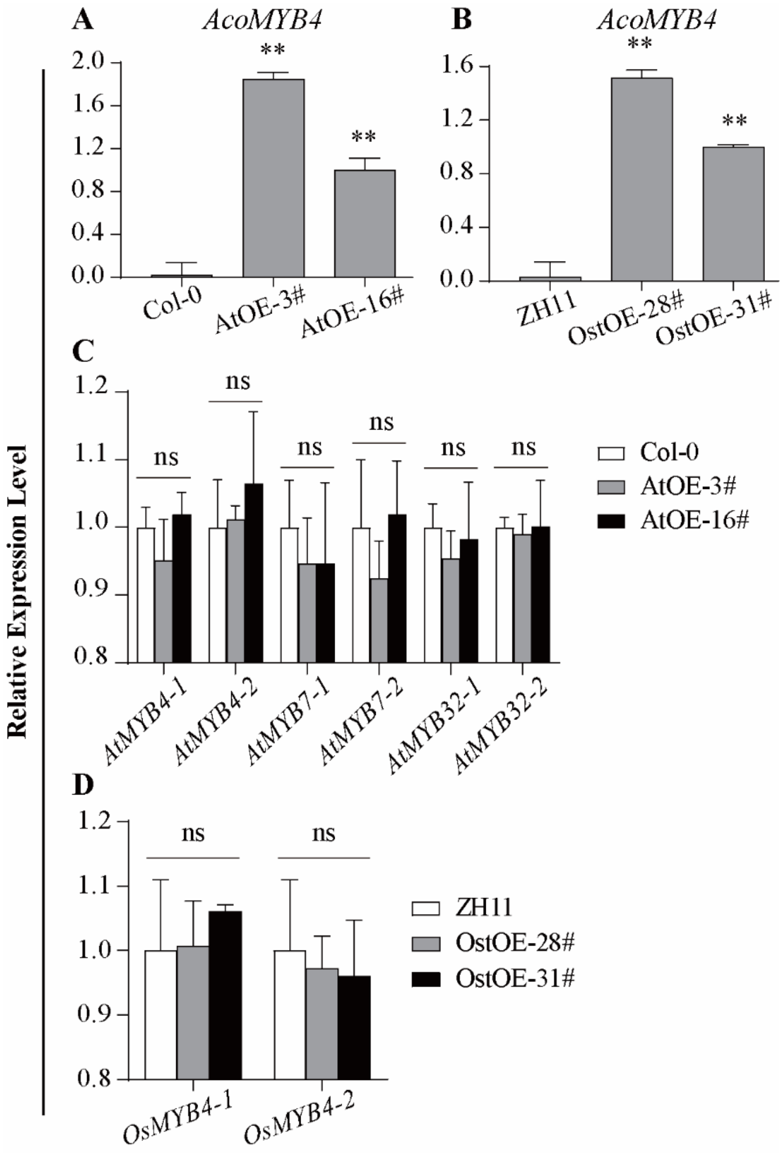

Supplemental Figure S1. Analysis of expression level of *AcoMYB4* and its homologous genes. (A) Relative expression level of *AcoMYB4* in Col-0, AtOE-3# and AtOE-16#. (The expression level of *AcoMYB4* in AtOE-16# is defined as 1). (B) Relative expression level of *AcoMYB4* in ZH11, OsOE-28# and OsOE-31#. (The expression level of *AcoMYB4* in OsOE-31# is defined as 1). (C) Relative expression level of *AtMYB4*(AtMYB4-1/-2), *AtMYB7*(AtMYB7-1/-2) and *AtMYB32*(AtMYB32-1/-2) in Col-0, AtOE-3# and AtOE-16#. (D Relative expression level of *OsMYB4* in ZH11, OsOE-28# and OsOE-31#. The error bars

indicate +SD (n=3). Asterisks indicate significant differences for the indicated comparisons based on a Students' t-test (** $p < 0.01$; * $p < 0.05$ and ns $p > 0.05$).

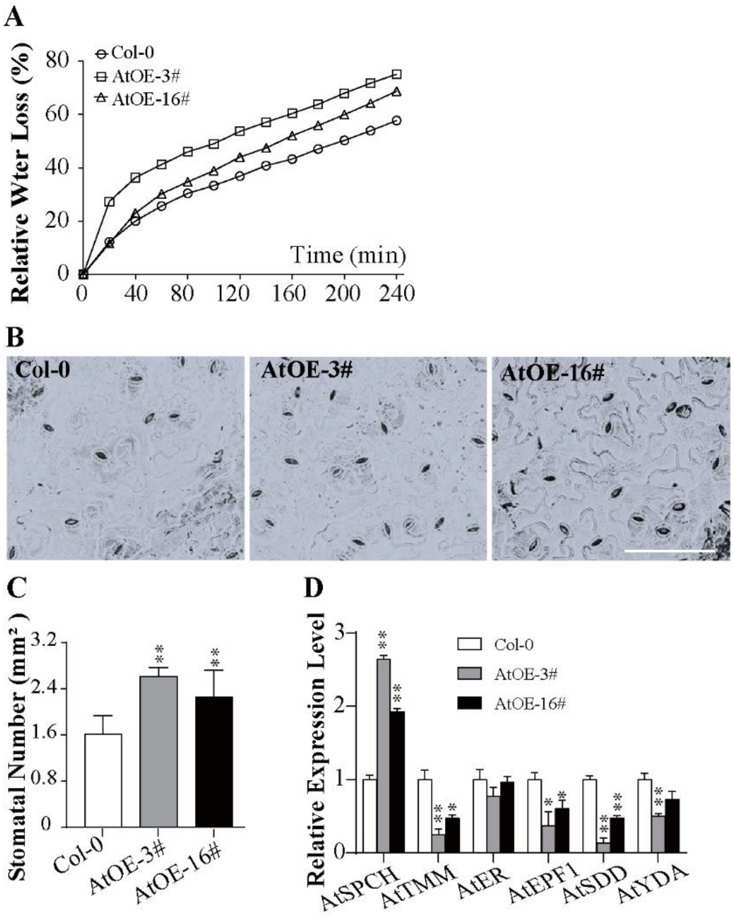

Supplemental Figure S2. Characteristics of stomatal phenotype of *AcoMYB4* overexpress *Arabidopsis* lines. (A) Relative water loss of detective leaves during a 240-min dehydration, the error bars indicate +SD (n=3). (C) Stomatal number and (B) representative image on the epidermis of underside leaves at 3-weeks-old seedling of AtOE-3#, AtOE-16# and WT, the error bars indicate +SD (n=20), Bar = 1 mm. (D) Relative expression level of *Arabidopsis* stomatal development related genes by q-PCR analysis, the error bars indicate +SD (n=3). Asterisks indicate significant differences for the indicated comparisons based on a Students' t-test (** $p < 0.01$; * $p < 0.05$).

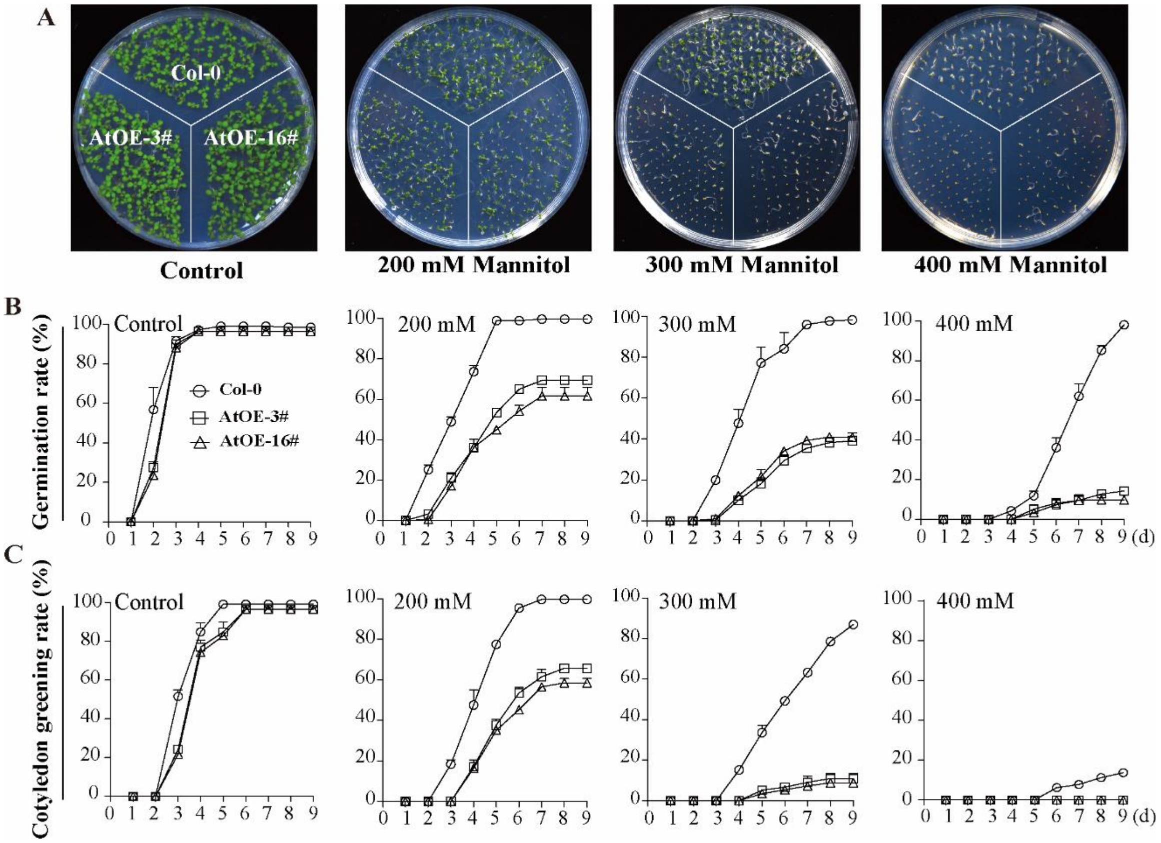

Supplemental Figure S3. Response of *AcoMYB4* overexpression plants seed germination and cotyledon greening rate in mannitol stress. Transgenic seeds were grown on media containing different mannitol concentrations. (A) Transgenic *Arabidopsis* (9th day) photographs of seedlings grown on different media at the end of stratification. Transgenic *Arabidopsis* seed germination rates (B) and cotyledon greening ratio (C), were quantified from the 1st to last day after sowing. The error bars indicate +SD (n=3 replicates).

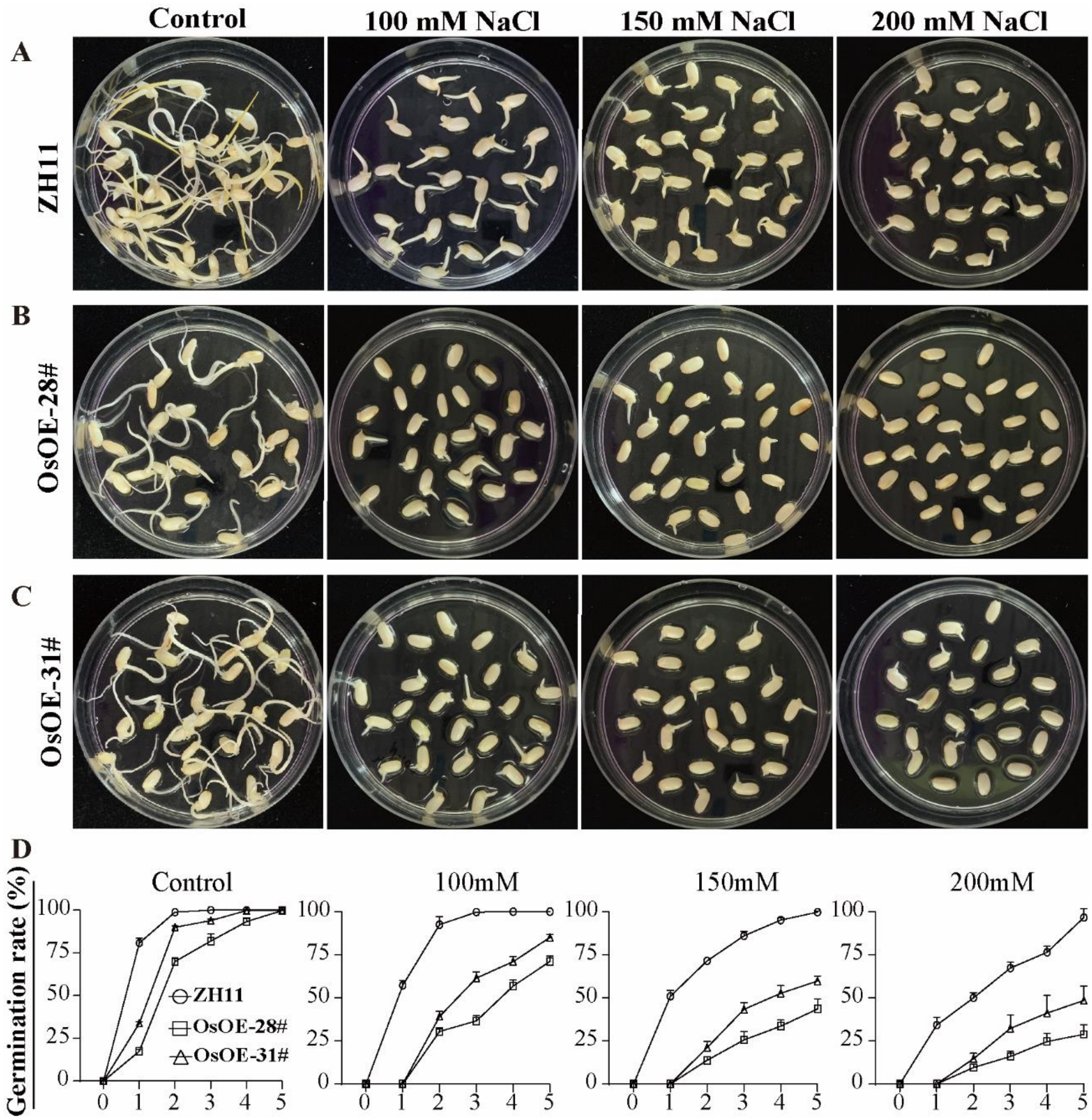

Supplemental Figure S4. ***AcoMYB4*** **overexpression lines seed germination rate under NaCl stress.** Transgenic rice seeds were grown on media containing different NaCl concentrations. Photographs show seedlings of transgenic rice (A, B and C; 5th day) grown on different mediums at the end of stratification. (D) Transgenic rice seed germination rates were quantified from the first to last day after sowing. The error bars indicate +SD (n=3 replicates).

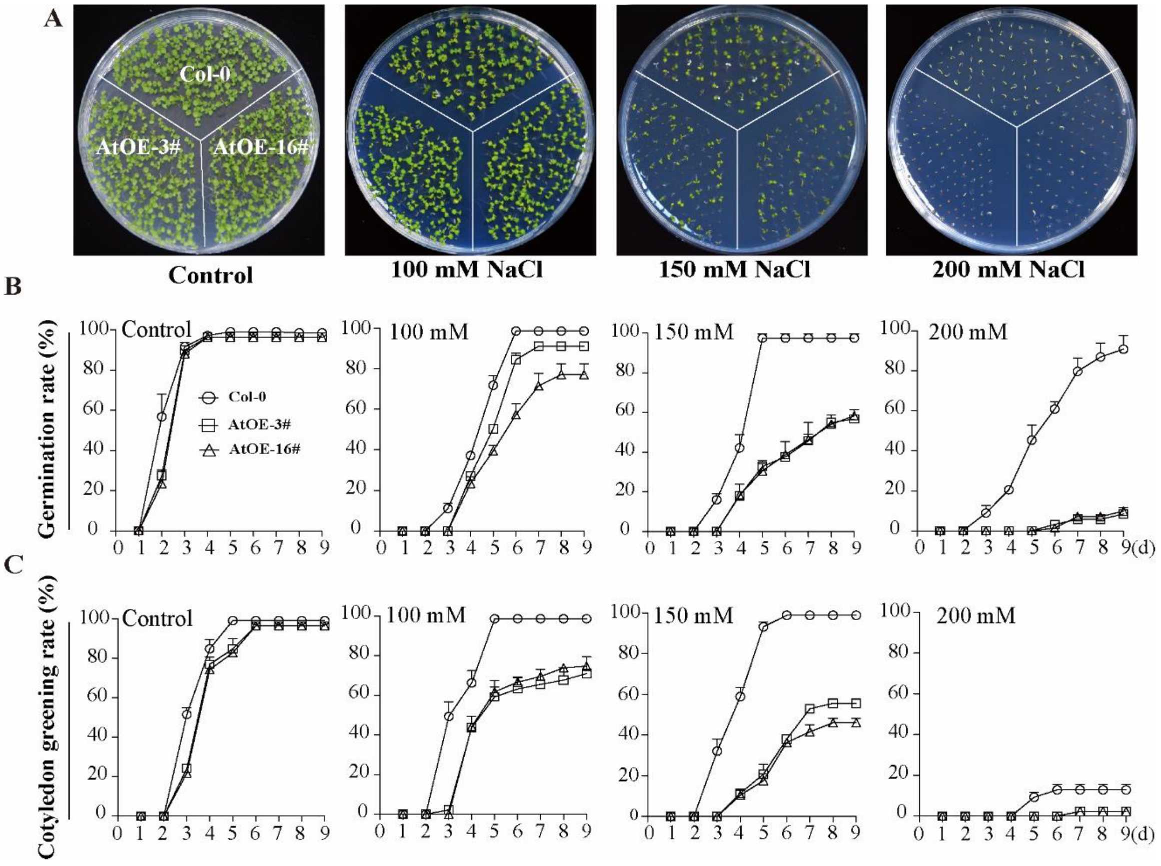

Supplemental Figure S5. ***AcoMYB4*** **overexpression lines seed germination and cotyledon greening rate under NaCl stress.** Transgenic seeds were grown on media containing different NaCl concentrations. (A) Transgenic *Arabidopsis* (9th day) photographs of seedlings grown on different media at the end of stratification. Transgenic *Arabidopsis* seed germination rates (B) and cotyledon greening ratio (C), were quantified from the 1st to last day after sowing. The error bars indicate +SD (n=3 replicates).

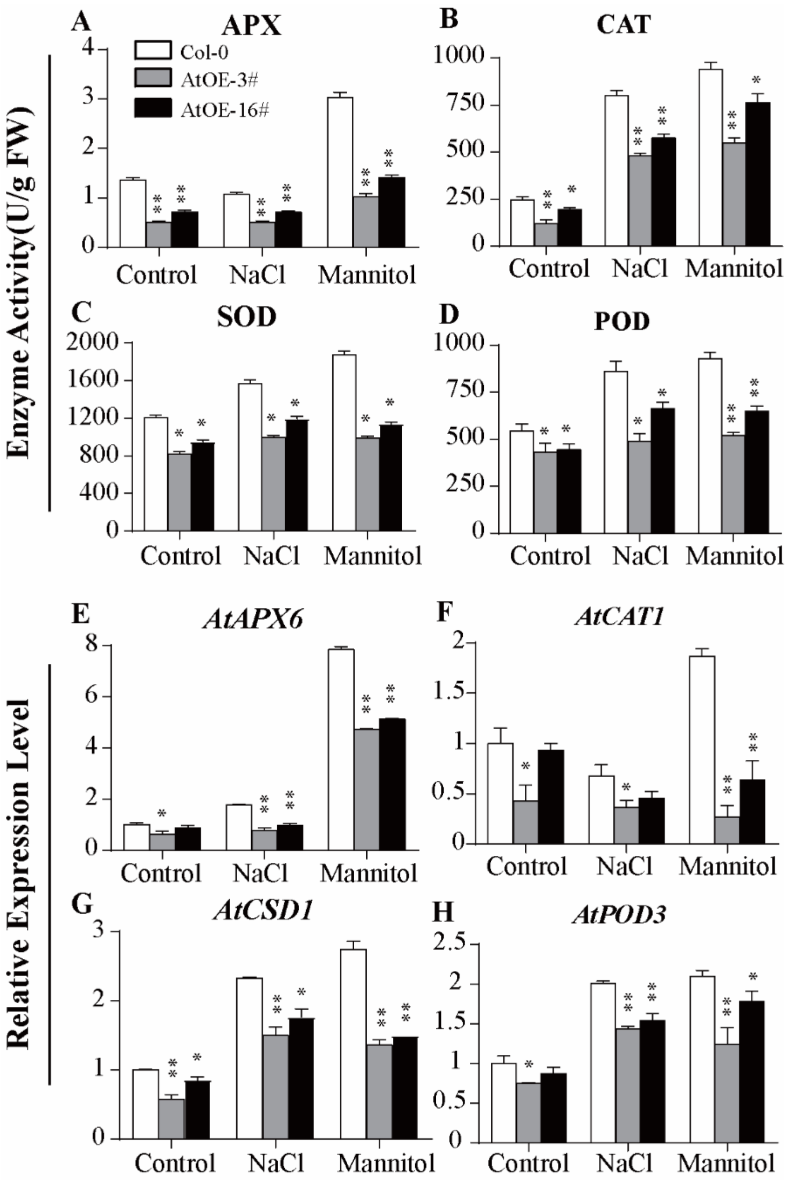

Supplemental Figure S6. ROS scavenges enzyme activities of *AcoMYB4* transgenic *Arabidopsis* lines under salt or drought condition. Three-week-old seedlings of AtOE-3#, AtOE-16# and WT were planted in the soil with or without 200mM NaCl or 400mM mannitol for 2 days. (A) APX, (B) CAT, (C) SOD and (D) POD enzyme activities were directly determined from fresh leaves. The relative expression level of (E) *AtAPX2*, (F) *AtCAT1*, (G) *AtCSD1* and (H) *AtPOD1* were analyzed by qRT-PCR. The error bars indicate +SD (n=3 replicates). Asterisks indicate significant differences for the indicated comparisons based on a Students' t-test (**$p < 0.01$; *$p < 0.05$).

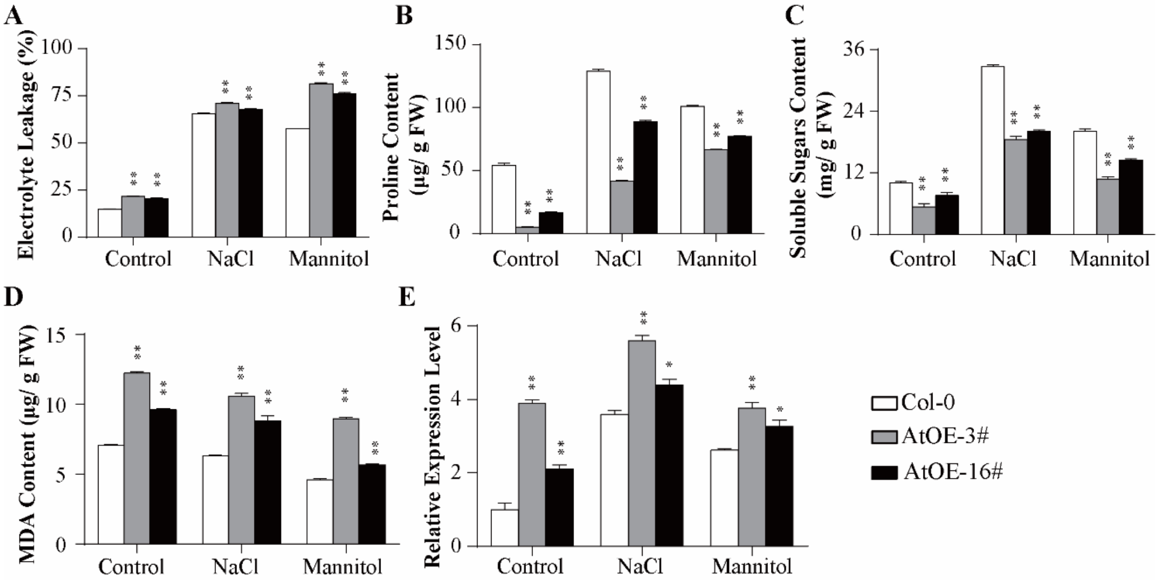

Supplemental Figure S7. Oxidative damage of *AcoMYB4* transgenic *Arabidopsis* lines under osmotic stress. (A) Electrolyte leakage, (B) Proline, (C) soluble sugars and (D) MDA content as well as (4) *AtMDA4* relative expression level in wild types and transgenic lines under salt or drought treatment for 2 days. The error bars indicate +SD (n=3 replicates). Asterisks indicate significant differences for the indicated comparisons based on a Students' t-test (**$p < 0.01$; *$p < 0.05$).

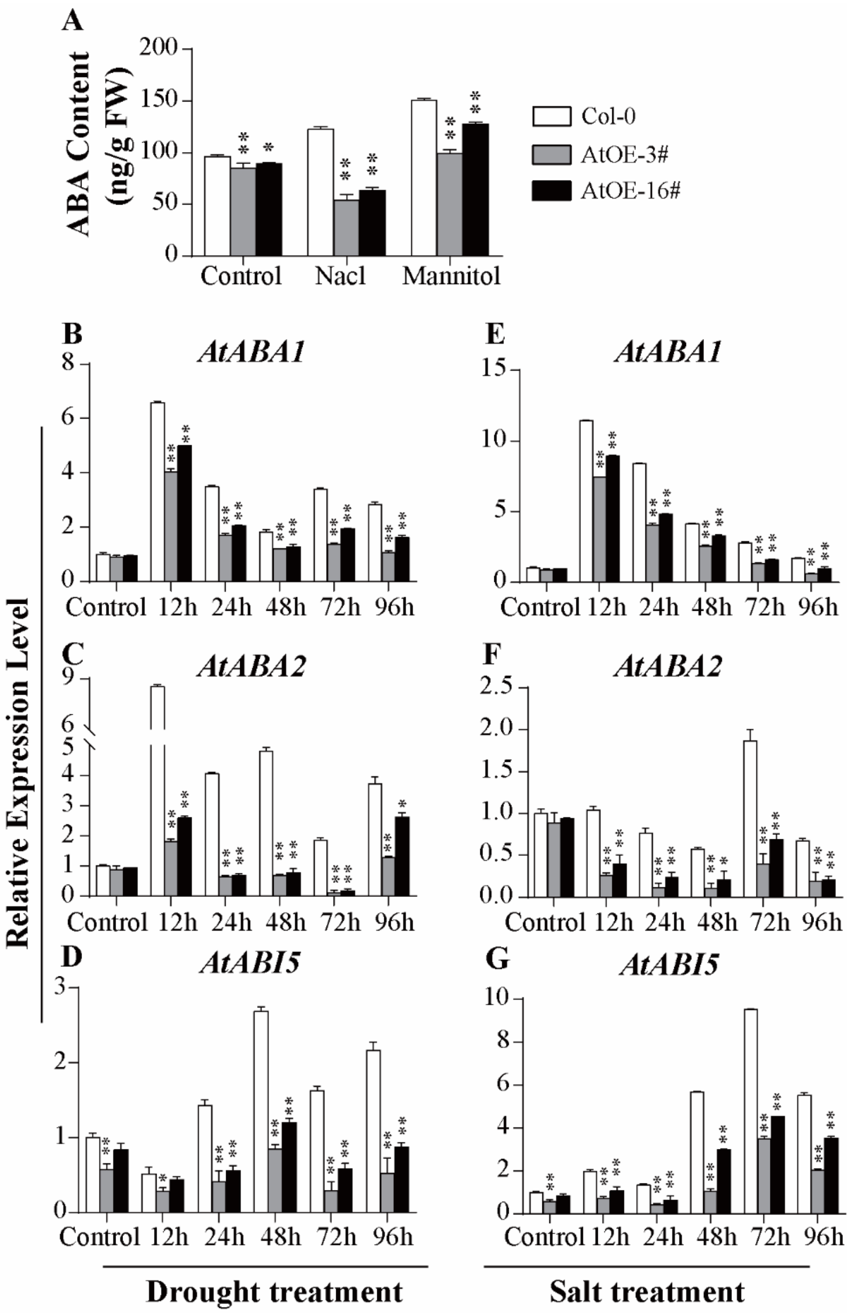

Supplemental Figure S8. Determination of ABA content and expression level of ABA signal pathway genes in transgenic *Arabidopsis* lines. (A) The endogenous ABA levels of transgenic rice lines and wild type leaves were determined by LC-MS under normal, salt and drought treatment for 48 h, respectively. The expression level of *AtABA1*, *AtABA2* and *AtABI5* in WT, AtOE-3# and AtOE-16# under (B-D) mannitol and (E-G) NaCl treatment. The error bars indicate +SD (n=3 replicates). Asterisks indicate significant differences for the indicated comparisons based on a Students' t-test (***p* < 0.01; **p* < 0.05).
