# Supplementary material for: AcoMYB4, an Ananas comosus L. MYB Transcription Factor, Functions in Osmotic Stress through Negative Regulation of ABA Signaling
Source: Int J Mol Sci. 2020 Aug 10;21(16):5727. doi: 10.3390/ijms21165727 (PMC7460842; doi:10.3390/ijms21165727)
Supplement: Supplementary file 1 [file ijms-21-05727-s001.zip › Supplemental filed/Supplemental Table S2.docx]

**Supplementary table S2. Synthetic information of *AcoABA1*, *AcoABI5*, *AcoABA1m* and *AcoABI5m* fragements.**

Gray background font marker extensions homologous to vector ends; yellow and green background font mark the first binding target site; green and blue background font together to form the second binding target site; red background font marker fragments with introduced mutations.

*AcoABA1*:

-547 ~ TCGAGCTCGGTACCCTTTTTTTTTCTTTTTTTTCCCCATCAACAAGTTTGGTAGGCGCTATACTGTGTGTGGATACAGCTAGATCGGGGATCTGTCGACC~ -477

*AcoABA1m*:

-547 ~ TCGAGCTCGGTACCCTTTTTTTTTCTTTTTTTTCCCCATCAACAACCACCAGTCAGTCTATACTGTGTGTGGATACAGCTAGATCGGGGATCTGTCGACC ~ -477

*AcoABI5*:

-833 ~ TCGAGCTCGGTACCCTATATCTATATATATTTTACTATTTCTTTTGTTGTTATAATTTATCATTCTATGCGTAAATTAGAATACTGGGGATCTGTCGACC ~ -763

*AcoABI5m*:

-833 ~ TCGAGCTCGGTACCCTATATCTATATATATTTTACTATTTCCCACCAGTCAGTTAATTTATCATTCTATGCGTAAATTAGAATACTGGGGATCTGTCGACC ~ -763
